# Supplementary material for: SERS, XPS and DFT Study of Xanthine Adsorbed on Citrate-Stabilized Gold Nanoparticles
Source: Sensors (Basel). 2019 Jun 15;19(12):2700. doi: 10.3390/s19122700 (PMC6631783; doi:10.3390/s19122700)
Supplement: Supplementary file 1 [file sensors-19-02700-s001.pdf]

SUPPLEMENTARY MATERIALS

SERS, XPS and DFT study of Xanthine adsorbed on citrate-stabilized gold nanoparticles

Stefano Caporali,[1] Francesco Muniz-Miranda,[2,#] Alfonso Pedone,[2] and Maurizio Muniz-Miranda [3,*]

[1] Department of Industrial Engineering, University of Florence, Via S. Marta 3, 50139 Firenze, Italy; stefano.caporali@unifi.it

[2] Department of Chemical and Geological Sciences, University of Modena and Reggio Emilia, Via Campi 103, 41125 Modena, Italy; f.muniz-miranda@chimieparistech.psl.eu (F.M.-M.); alfonso.pedone@unimore.it (A.P.)

[3] Department of Chemistry "Ugo Schiff", University of Florence, Via Lastruccia 3, 50019 Sesto Fiorentino, Italy

* Correspondence: maurizio.muniz@unifi.it; Tel.: +39-349-184-9350

# current address: Chimie ParisTech, PSL Research University, CNRS, Institute of Chemistry for Life and Health Sciences, F-75005 Paris, France.

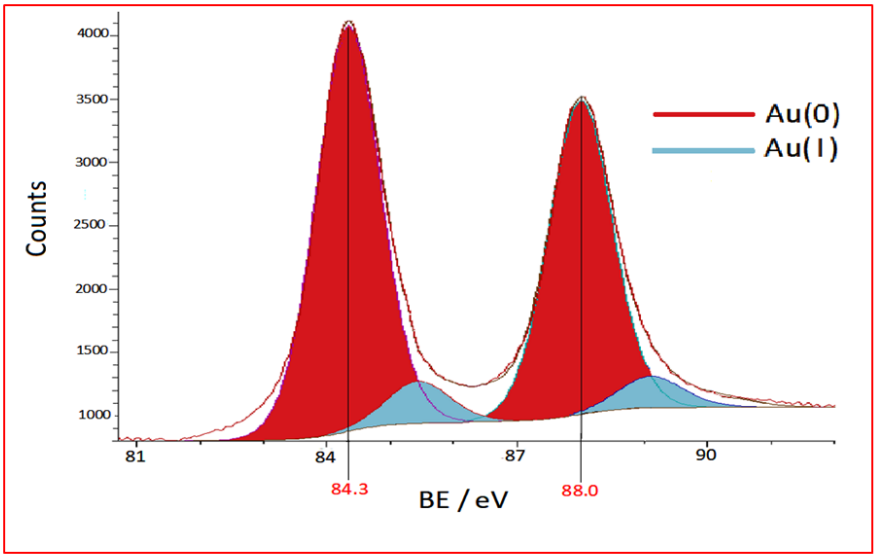

Figure S1. XPS spectrum of gold nanoparticles obtained by nanosecond pulsed laser ablation in pure water.

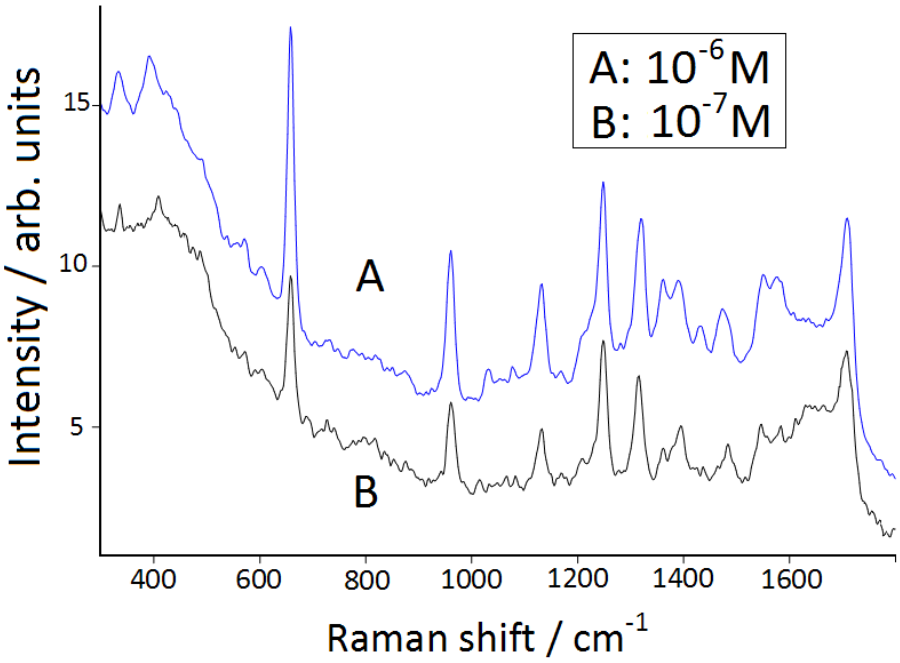

Figure S2. Comparison between the SERS spectra of xanthine at two different ligand concentrations.

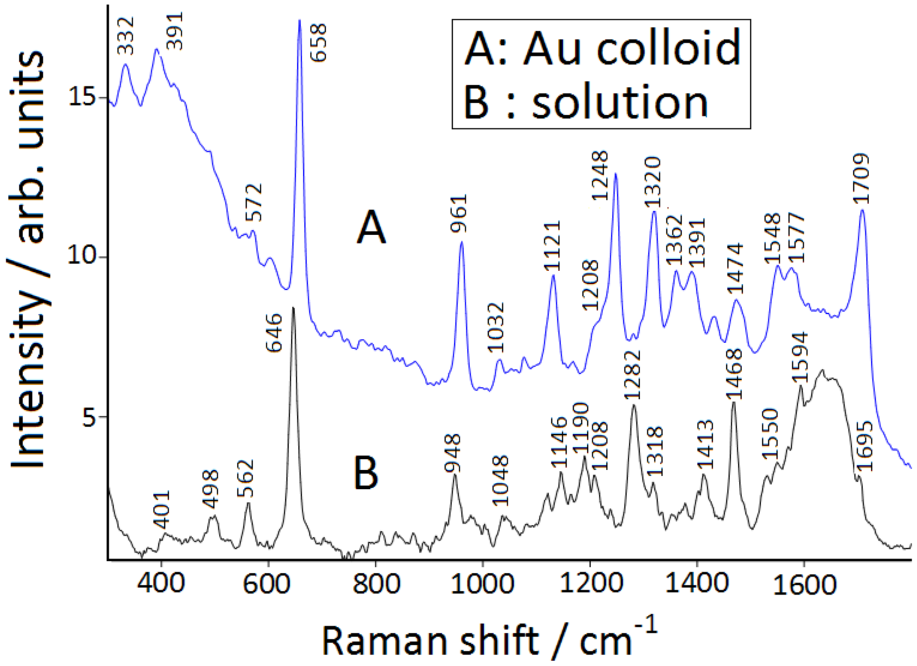

Figure S3. Comparison between the SERS spectrum of xanthine (10^{-6}M concentration) and the Raman spectrum of xanthine dissolved in water (10^{-3}M concentration). Intensities of the Raman spectrum multiplied by ten.

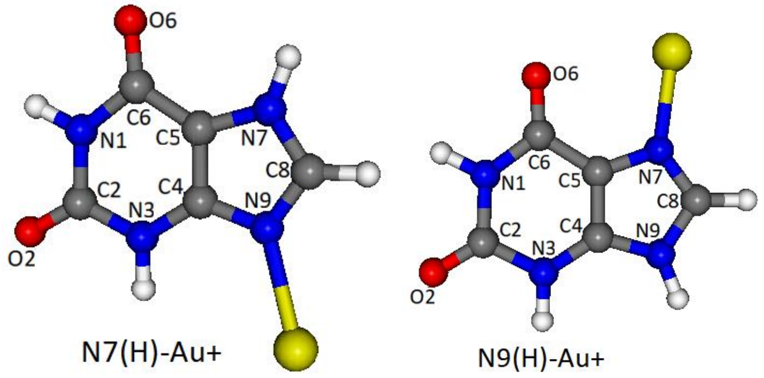

Table S1. Atomic partial charges in the N7(H)-Au^+ and N9(H)-Au^+ complexes.

	Mulliken partial charges		Hirshfeld partial charges	
Atom	N7(H)-Au+	N9(H)-Au+	N7(H)-Au+	N9(H)-Au+
N1	0.006436	0.002789	0.109841	0.117140
C2	0.211402	0.209670	0.227629	0.231632
N3	-0.024762	0.003478	0.092833	0.119341
C4	0.358532	0.283899	0.094217	0.113716
C5	0.122840	0.179601	0.007398	-0.017308
C6	0.197987	0.272008	0.177419	0.176011
O2	-0.203904	-0.196826	-0.268161	-0.260167
O6	-0.213755	-0.257255	-0.261522	-0.267919
N7	0.071558	-0.406832	0.216483	-0.064948
C8	0.211402	0.256241	0.227629	0.210997
N9	-0.505563	0.003478	-0.078160	0.177603
Au	0.672052	0.645610	0.469171	0.463860

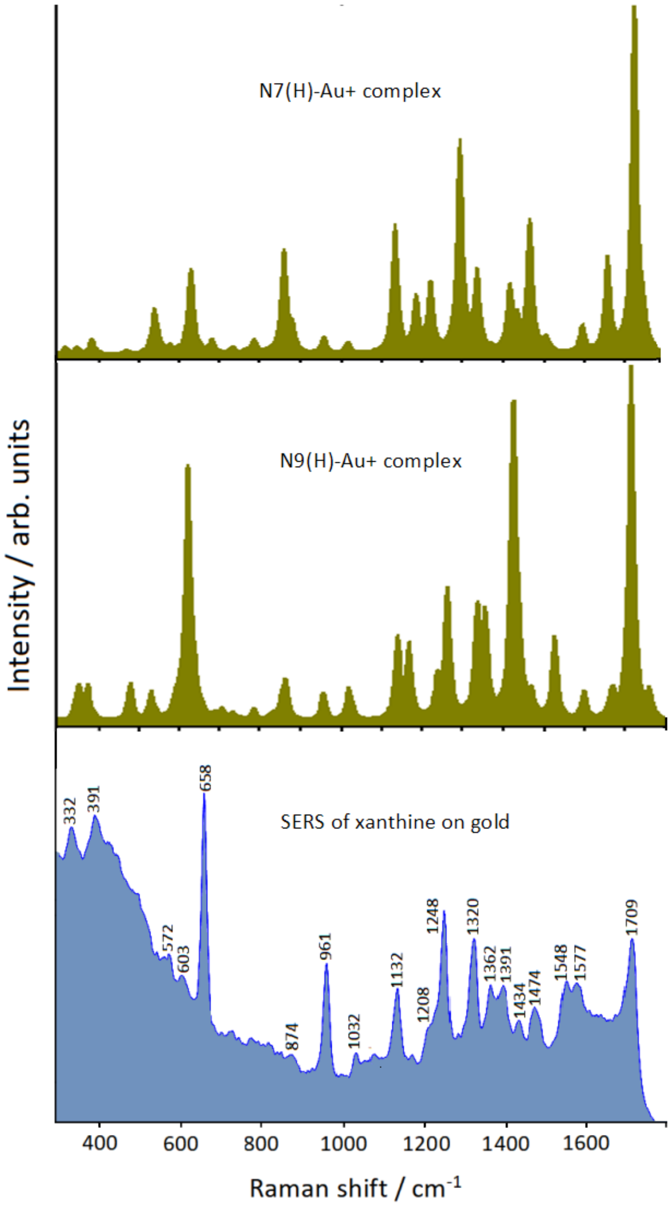

Figure S4. Comparison between simulated and observed SERS spectra.

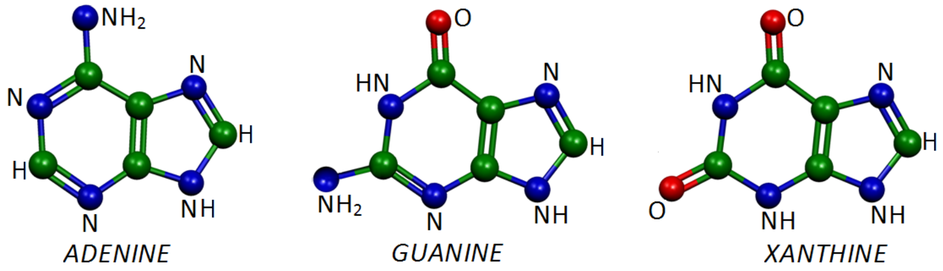

Table S2. Marker SERS bands of purine nucleobases adsorbed on gold.

ADENINE [a]	GUANINE [b]	XANTHINE [c]
630 m		
	665 vs	658 vs
735 vvs		
	958 s	961 s
	1222 m	
		1248 s
1323 m		1320 s
1342 m	1352 s	
1450 m		
	1705 s	1709 s

[a] refs. 36,37; [b] ref. 38; [c] see Figure 6.
